# Knowledge, attitude, and practice among mothers about newborn care in Sindh, Pakistan

**DOI:** 10.1186/s12884-019-2479-0

**Published:** 2019-09-06

**Authors:** Javed Memon, Kourosh Holakouie-Naieni, Reza Majdzadeh, Mir Saeed Yekaninejad, Gholamreza Garmaroudi, Owais Raza, Shahrzad Nematollahi

**Affiliations:** 1 School of Public Health, International Campus, Tehran University of Medical Sciences, Tehran, Iran; 2 Department of Epidemiology and Biostatistics, School of Public Health, Tehran University of Medical Sciences, Tehran, Iran; 3 Department of Epidemiology and Biostatistics, School of Public Health, Tehran University of Medical Sciences, Tehran, Iran; 4 Department of Health Education & Promotion School of Public Health, Tehran University of Medical Sciences, Tehran, Iran; 5 Men`s Health & Reproductive Health Research Center, Shahid Beheshti University of Medical Sciences, Tehran, Iran

**Keywords:** Knowledge, Attitude, Practice, Newborn care

## Abstract

**Background:**

Each year nearly 7.7 million children under five years die around the world; out of which approximately 3.1 million of the newborns die during the neonatal period and almost all these (99%) deaths occur in the developing countries. According to the World Health Organization’s estimation neonatal deaths account for 45% of the under-five deaths. More than one-third of these deaths occur in the first 24 h of birth, whereas three-quarter of the neonatal deaths takes place in the first seven days of birth. The purpose of this study is to assess the knowledge, attitude, and practices (KAP) among mothers about newborns’ care and its related factors in district Badin Sindh province of Pakistan.

**Methods:**

A community-based cross-sectional study was conducted from July 2017 to August 2017 to assess the Knowledge, Attitude, and Practices (KAP) in mothers regarding newborn care. A structured questionnaire was administered, after pretest, for data gathering through face to face interview. All survey participants were identified using multi-stage cluster sampling. A scoring system was used to calculate the level of KAP among participants. Independent sample t-test, ANOVA, and GLM were applied to identify the statistical difference between the means of various groups.

**Result:**

A total of 518 survey participants were interviewed. Among the study sample, more than half of the newborns were bathed within six hours of delivery. Around 50% started breastfeeding after 1 h of birth. A substantial proportion (45%) of mothers gave pre-lacteal feeding and 44.8% of them did not feed colostrum to their newborns. Among those who administered pre-lacteal to their newborn babies included animal/formula milk (15.4%), honey (24.5%) and fresh butter/ghee (5.2. %). Mothers with no education had less significant KAP score about newborn care as compared to those who had higher education (*p* < 0.05).

**Conclusion:**

This study revealed that high-risk factors such as immediate bathing, application of traditional substances on the cord, delayed initiation of breastfeeding, discarding colostrum and giving pre-lacteal feed to newborns were highly prevalent. This requires urgent attention of Maternal, Newborn and Child Health (MNCH) programs and health care delivery system to prevent harmful care practices and adopt healthy practices especially in the rural settings.

## Background

Each year nearly 7.7 million children under five years die around the world; out of which approximately 3.1 million of the newborns die during the neonatal period and almost all these (99%) deaths occur in the developing countries [1]. According to the World Health Organization estimation, neonatal deaths account for 45% of the under-five deaths [2]. More than one-third of these deaths take place in the first 24 h of birth, whereas three-quarter of the neonatal deaths takes place in the first seven days of birth [3, 4].

Among the17 Sustainable Development Goals (SDGs) set by United Nations in 2015, the 3rd goal, target (No. 3.2) states that all countries aim to put a stop to millions of avoidable deaths of newborns and under-five children by 2030. The targets to achieve are: reduction in neonatal mortality and under-5 mortality to no more than 12 and 25 deaths per 1000 live births respectively [5]. Majority of low-income countries are far behind achieving this goal mainly because of slow progress in reducing neonatal death [6].

In Pakistan, despite the reduction in under-five and infant mortality rate, there is almost no change in neonatal mortality for the last two decades (from 56 to 46 per 1000 live births) [7]. Pakistan ranks third among the top ten countries with high incidence of neonatal deaths [8]. At these mortality levels, 1 in every 14 Pakistani children is unable to survive before the first birthday, while 1 in every 11 dies before the age of five [9]. The increasing evidence suggests that early newborn care practices impact neonatal mortality and morbidity. The burden of neonatal mortality and morbidity can be reduced by practicing essential newborn care (ENC) practices [10]. Many studies have been done on the newborn care topics in Pakistan and in developing countries but most of them only focus on newborn care practices [11–14].

In Pakistan, mothers are the primary caregiver to newborns hence the care is mostly dependent on their level of knowledge, attitude, and practice (KAP) about newborn care. Therefore, this study aims to investigate the KAP of mothers about newborn care and its related factors to achieving optimum newborn care.

## Methods

### Study design, area and period

To investigate the research topic, a community based cross-sectional study was performed in Badin, a rural district of province Sindh in Pakistan. The total area of this district is 6527 km^2^. It is divided into five sub-districts namely Badin, Matli, TandoBago, Golarchi, and Talhar. The sub-districts are further administratively divided into 46 union councils. The district has total population 1,917,822. The population density of the district is 285 people per square kilometer and an estimated annual growth rate of the district is 2.26%. Sex ratio in Badin district is 111 male per 100 females, which is more than the ratio at the national level that is 106. Out of the total population, 53% are males and 47% are females. 84% of the population resides in a rural area as compared to the 16% that resides in the urban areas. The main language spoken in Badin district is Sindhi [15]. This study was conducted from June 2017 to July 2017.

### Study population

The Study population consisted of married women of reproductive age ranging between 15 and 49 years, having at least one infant up to 12 months and residing in the locality for at least a year where the study was being conducted. Since it was an observational study for a limited period of time without any intervention; therefore an exception to the written consent was given instead verbal consent was taken from participants. However, for only two participants written consent was taken from their parents who were below the age of 16.

### Sample size determination and sampling technique

A sample size of 528 was calculated with the following assumptions: considering 95% confidence interval, standard deviation 30 (taken from the pilot study), 5% precision, 10% non-response rate, and design effect 2.

In order to select a study unit, multi-stage cluster sampling was used. For stage one, three out of five sub-districts of Badin were selected randomly. At stage two, via proportionate sampling, allocation of the sample size for each selected sub-district was determined proportionally to the number of households within each sub-district. For the third stage, the total number of the households was divided over the sample size to give sampling interval (k) then participants were selected based on every kth household using a systematic sampling technique. In cases where more than one mother was present in the same household, only one of them was randomly selected for an interview.

#### Data collection tools and processes

First questionnaire was developed in the English language to ensure the data quality assurance and then it was translated into the local language (Sindhi) of the study area by professional to make the questions easily understandable to the study population and it was retranslated back into the English language to evaluate its consistency. Data collection was conducted through individual interviews by trained data collectors using a pre-tested structured questionnaire. This questionnaire consisted of four parts; the first part collected socio-demographic details of the participants (such as age, education, occupation, residence etc.), whereas the rest three sections inquired the level of KAP (Knowledge, Attitude and Practices) regarding newborn care on thermoregulation, umbilical cord care, and breastfeeding. The knowledge section consisted of 11 questions; attitude section had 5 while practice section included of 9 questions.

A scoring system based on the World Health Organization (WHO) essential newborn care guidelines was used to analyze responses on KAP sections [16]. Each correct answer was assigned “1 score” and wrong answer was given “0 score”. For the Knowledge scale, the scores ranged from 0 to 11 points, where for Attitude, scores ranged from 0 to 5, while for Practice scale, the scores ranged between 0 and 9 points.

### Validity and reliability procedure

The questionnaire was adopted from WHO tool [16], accordingly from previous studies [11, 13, 17, 18] and after consultation with an experts in public health to include their opinions regarding the construct of the questionnaire to ascertain the validity. Required modifications were done based on the outcome variable of the study.

In order to check the reliability, a pilot study was conducted on the 30 participants. The Cronbach’s alpha coefficient values for knowledge, attitude and practice items were 0.75, 0.62 and 0.72 respectively.

### Statistical analysis

After data collection was completed, the data was checked for any errors or incomplete information, so that it could be excluded from the entry. After data cleaning, the data was entered into Microsoft Excel and analyzed using STATA version 14 software. Univariate analysis was used for descriptive statistics such as frequencies, percent distribution, means and standard deviation to describe socio-demographic characteristics of the sample mothers.

Socioeconomic status (SES) of the respondents was assessed on the basis of their household’s assets by using Principal Component Analysis (PCA) method. The resulting index was divided into three categories of ‘Poor’, ‘Average’ and ‘Rich’. This socioeconomic status serves as the proxy indicator of household wealth that has been consistent with household income.

As for the bivariate analysis, independent sample t-test and ANOVA were applied to identify a statistical difference between the means of various groups. In order to compare mean scores of KAP about newborn care across the groups with more than two categories, a Scheffe post-hoc analysis was performed. For multivariate, analysis, the generalized linear model (GLM) was used to identify independent factors associated with the KAP of newborn care after making an adjustment for other variables.

## Results

The sample consisted of 528 participants and had an overall response rate of 98.1%. The mean age of the mothers was 28.8 ± 5.8 years. 55.5% of the participants belonged to the age range of 20–29 years. Around 66% of the participants resided in the rural areas and 53% of respondents lived in joint families. 64.9% had no education at all, whereas only 14.1% studied up to secondary level. 66% of these women were housewives while 34% had outdoor occupations. 20% of the working mothers were farmers. (Table 1).

**Table 1:** Percent mean score of knowledge, attitude and practice among mothers regarding newborn care and the result of adjusted analysis of the effect of independent variables. Participants’ scores of KAP are calculated out of 100

Variable	Category	Frequency		Knowledge				Attitude				Practice			
		*n*	%	Mean (95% CI)	P-crude	Adjusted		Mean (95% CI)	P-crude	Adjusted		Mean (95% CI)	P-crude	Adjusted	
						Beta (SE)	P val			Beta (SE)	P val			Beta (SE)	P val
Age(Y)	14–19	14	2.7	38.0(26.6–50.0)	0.001	−0.0;.5.6	0.995	42.9(28.6–57.1)	0.002	−9.7;7.5	0.194	46.0(37.7–54.3)	0.001	11.9;6.6	0.071
	20–29	288	55.5	51.2(49.5–54.1)		9.0;.3.7	0.014	64.0(61.4–66.7)		5.9;4.9	0.224	52.7(50.1–55.2)		14.5;4.3	0.001
	30–39	195	37.6	49.0(46.6–51.3)		10.2;.3.7	0.039	63.3(60.1–66.7)		7.6;4.9	0.128	51.0(48.1–54.0)		16.6;4.4	0.000
	> 40	21	4.0	38.3(31.2–44.9)		1	NA	54.3(45.6–63.0)		1	NA	33.3(25.7–41.0)		1	NA
Education	No education	337	64.9	43.1(41.3–44.9)	0.001	−14.3;2.9	0.001	57.9(55.6–60.2)	0.001	−14.6;3.9	0.000	44.9(42.8–47.0)	< 0.001	− 14.4;3.4	< 0.001
	Primary	63	12.1	53.4(48.8–58.0)		−7.3;3.1	0.020	60.6(55.1–66.2)		−14.2;4.1	0.001	52.6(47.2–57.9)		−9.6;3.6	0.008
	Middle	45	8.7	63.9(58.3–67.8)		−3.4;3.1	0.265	71.1(64.5–77.7)		−8.7;4.1	0.032	64.9(59.1–70.8)		−1.8;3.6	0.601
	Higher&above	73	14.1	70.0(66.9–73.1)		1	NA	81.9(77.6–86.2)		1	NA	69.9(66.8–73.0)		1	NA
Occupation	House wife	342	65.9	47.0(45.0–49.1)	0.001	−2.2;1.8	0.217	60.5(58.1–62.8)	0.001	1.2;2.4	0.624	49.3(47.0–51.4)	< 0.001	0.6;2.1	0.759
	Private	31	6.0	63.0(56.1–70.0)		0.9;3.4	0.795	72.3(63.6–80.9)		2.1;4.5	0.643	63.1(55.3–70.9)		1.7;3.9	0.674
	Governmental	40	7.7	65.5(61.4–69.5)		0.5;3.1	0.882	81.0(75.2–86.8)		9.2;4.2	0.028	65.6(61.2–70.0)		1.7;3.7	0.636
	Farmer	105	20.2	49.2(45.5–52.8)		1	NA	60.6(56.1–65.0)		1	NA	48.0(43.9–52.2)		1	NA
Ethnicity	Sindhi	396	76.3	47.8(46.0–49.5)	0.001	2.8;1.9	0.155	60.5(58.3–62.7)	< 0.001	−1.5;2.6	0.564	49.7(47.7–51.7)	< 0.001	5.3;2.8	0.020
	Mohajir	35	6.3	71.0(65.9–75.9)		8.3;3.3	0.012	78.3(71.9–84.6)		3.5;4.4	0.428	69.2(64.0–74.5)		6.8;3.8	0.078
	Siraki,others	86	20	49.9(46.2–55.9)		1	NA	66.9(61.3–72.6)		1	NA	50.1(44.8–55.4)		1	NA
Residence	Urban	178	34	62.1(59.7–64.6)	.001	7.6;2.0	0.001	70.0(66.6–73.4)	0.01	−1.3;2.7	0.619	62.7(60.0–65.3)	0.01	6.9;2.3	0.003
	Rural	340	65.9	43.4(41.6–45.3)		1	NA	59.0(56.7–61.3)		1	NA	45.0(42.8–47.2)		1	NA
Family system	Joint	275	53.0	48.5(46.2–50.8)	.088	NA	NA	63.0(60.4–65.5)	0.831	NA	NA	51.5(49.1–53.9)	0.63	NA	NA
	Nuclear	243	47	51.4(48.9–53.8)		NA	NA	62.6(59.5–65.6)		NA	NA	50.6(47.8–53.5)		NA	NA
Socio Economic Status	Poor	173	33.4	62.4(59.8–65.1)	.001	5.2;2.3	0.021	73.4(70.2–76.6)	0.001	6.8;3.0	0.024	63.1(60.3–65.9)	< 0.01	6.3;2.6	0.017
	Average	172	33.2	44.0(41.6–46.4)		−0.5;1.7	0.784	57.3(54.1–60.5)		−1.8;2.3	0.439	45.5(42.5–48.5)		−0.2;2.0	0.933
	High	173	33.4	43.1(40.3–45.8)		1	NA	57.6(54.2–61.0)		1	NA	44.6(41.6–47.7)		1	NA
Child Sex	Male	261	28.1	48.7(46.4–51.0)	0.2	NA	NA	63.8(61.0–66.6)	0.2	NA	NA	49.3(46.7–51.9)	0.063	NA	NA
	Female	257	39.1	51.0(48.6–53.5)		NA	NA	61.7(58.9–64.5)		NA	NA	52.1(50.3–55.4)		NA	NA

P-Crude is from ANOVA and or t-test. Adjusted results from GLM. (see text). Beta is the constant value of the GLM. NA: Not applicable

The mean KAP scores for these mothers were 5.48 (out of 11) for the scale of Knowledge, 3.14 (out of 5) for the scale of Attitude and 4.60 (out of 9) for the scale of Practice.

Respondents’ knowledge varied for different aspects of newborn care as per WHO guidelines; 57% of the participants had correct knowledge regarding skin to skin contact, whereas 55% knew about the correct timing of newborn’s first bath, 54.6% had accurate knowledge about initiation of breast milk, 57.6% knew about pre-lacteal feed and 55.6% of the participants could respond correctly about giving colostrum to their newborns. Only 1.4% of the subjects informed that chlorhexidine should be applied on the umbilical cord and the rest reported that any type of oil, dettol, common powder, and antimony could be used. Around 73.4% of study subjects knew that breastfeeding should be given on demand. More than half of the participants 51.0% were aware about six months duration of exclusive breastfeeding. (Table 2).

**Table 2:** Distribution of respondents by knowledge, attitude and practices among mothers about newborn care

Parameter	Knowledge N (%)	Attitude N (%)	Practice N (%)
Skin to skin contact			
Yes	295(57.0)	308(59.5)	296(57.1)
No	223(43.0)	210(40.5)	222(42.9)
Time of first bathing			
Within 6 h	223(45.0	316(61.0)	268(51.8)
After 6 h	185(55.0)	202(39.0)	250(48.2)
Instrument used to cut the cord			
Used blade	17(3.3)	60(11.5)	15(2.9)
New blade	353(68.1)	458(88.4)	485(65.4)
Scissor	148(28.6)	NA	164(31.7)
Material application to cord			
Any type of oil	35(6.8)	NA	32(6.2)
Dettol/Spirit	354(68.3)	NA	371(71.6)
Common powder	78(15.1)	NA	60(11.6)
Chlorhexidine	7(1.4)	NA	8(1.5)
Antimony	44(8.5)	NA	47(9.1)
Initiation of breast feeding			
Within 1 h	283(54.6)	NA	255(49.2)
After 1 h	235(45.4)	NA	163(50.8)
Prelacteal feeding			
Yes	220(42.4)	165(31.8)	234(45.2)
No	298(57.6)	353(68.1)	284(54.8)
Type of prelacteal feeds			
Honey/Sugar	138(26.6)	NA	127(24.5)
Fresh butter/Ghee	25(4.8)	NA	27(5.2)
Milk (other than breast milk)	57(11.1)	NA	80(15.4)
Colostrum feeding			
Feed the baby	288(55.6)	305(58.9)	286(55.2)
Throw it away	230(44.4)	213(41.1)	232(44.8)
Breastfeeding on demand (when baby cries/looking for breast)			
Yes	380(73.4)	NA	337(65.1)
No	138(26.6)	NA	181(34.9)
Know about exclusive breastfeeding			
Yes	283(54.6)	NA	NA
NO	235(45.4)	NA	NA
Duration of Exclusive breastfeeding			
Less than 6 months	149(28.7)	NA	NA
For 6 months	264(51.0)	NA	NA
Greater than 6 months	105(20.3)	NA	NA

N (%): *NA* Not applicable

Study participants were assessed on their attitude about newborn care, out of total sample unit, 59.5% perceived that skin to skin contact is a preventive method from getting cold to a baby. Most of the respondents 61.0% did not accept delaying bathing of their newborns babies. Majority of the participants 88.4% preferred to use a new blade for cord cutting. Around 68.1% believed that prelacteal feeding should not be administered and 41.1% had a negative attitude on regarding colostrum feeding as shown in Table 2.

More than half of the respondents stated that they bathed their newborns within six hours of delivery. Around 50% of subjects breastfed newborns after one hour of their birth. 44.8% stated that they did not feed colostrum to their newborns. Approximately 45% of the mothers reported that they gave pre-lacteal feed to their newborns. Among these, 15.4% of respondents gave animal/formula milk, 24.5% gave honey/sugar and 5.2% gave fresh butter/ghee. Majority of mothers 65.1% breastfed their babies on demand. (Table 2).

### Knowledge

The significant difference in the mean score was observed between knowledge and age (*p* = 0.001). Post hoc analysis result showed that mothers belonging to the age group of 14–19 had significantly lesser knowledge score (*p* = 0.049) as compared to the ones above 19. The knowledge score was statistically different among education level groups (*p* = 0.001). The smallest score (43.1) associated with the no education while highest score (70.0) with the higher level group. According to post-hoc analysis, mothers with no education had significantly lower score compared to those with education (*p* < 0.001). However, there was no such difference seen in the knowledge score for the mothers with middle and higher education (*p* = 0.117). The mean score of knowledge in occupation groups was seen significantly different (*p* = 0.001). The respondents who were government employees had the maximum score (65.5) whereas housewives had the lowest (47.0). Respondents who were housewives scored lowest compared to other categories (p = < 0.001). But the difference was not significant when the scores of housewives and those working as farmers were compared (*p* = 0.730). Moreover, the difference between knowledge mean score and ethnicity was significantly visible (*p* = 0.001). The highest score (71.0) was in *Mohjar* and the smallest (47.8) in *Sindhi* group. The difference between the residence type and knowledge score was significant (p = 0.001). The people living in urban had a higher score (62.1) as compared to those who were inhibiting in rural areas (43.4). The knowledge mean score was not significantly detected in the family type (*p* = 0.088) and sex of the child (*p* = 0.2). (Table 1).

### Attitude

A significant difference was observed in age group (*p* = 0.002). The age group 14–19 had a lower score than others. The Following age group 20–29 had a statistically higher score than 14–19 group (*p* = 0.009). Education level, occupation, ethnicity, residence type were highly significantly different to the attitude score (*p* < 0.001). Participants belonging to higher education, Government employee, *Mohajir* and urban categories had more attitude score on newborn care than the others. However, there was no significant difference between attitude mean score and family type (*p* = 0.831) and sex of the child (*p* = 0.2). (Table 1).

### Practice

The practice mean score in maternal age was significantly different (*p* = 0.001). The oldest age group > 40 had the lowest percentage mean practice score than the rest. However, the highly significant difference was observed between the practice score and education (*p* < 0.001). In the post hoc analysis, there was a significant difference between no education and the rest (*p* < 0.001). But the difference between middle and higher education groups was not significant (*p* = 0.176). The practice score was different among occupation categories (p < 0.001). The housewife had a significantly lower score than a private employee (*p* = 0.006). There was no significant difference between the farmer and housewife groups (*p* = 0.956). Place of residence was highly associated (*p* = 0.01). The significant difference in practice mean score was not detected to family type (*p* = 0.63) and sex of the child (*p* = 0.054). (Table 1).

### Determinants

Mothers aged between 20 to 29 had a significantly higher mean score of knowledge as compared to those respondents who were aged forty and above (B = 9.0; *p* = 0.014). Women with no formal education had lesser knowledge score compared to the women who had higher education (B = -14.3; *p* = 0.001). Occupation of the respondents did not show any significant effect on the knowledge score (*p* > 0.05). Mothers who were *Mohajirs* had better mean knowledge score than those who were *Siraiki* or belonged to other ethnicities (B = 8.3; *p* = 0.012). Those who were living in the urban areas had a higher mean score for knowledge than who were residing in rural areas (B = 7.6; *p* = 0.001).

A significant relation was found between the levels of education and attitude score of the participants. Mothers with no education had less attitude score towards newborn care than participants who had higher or more education (B = -14.6); *p* = 0.000).

The practice score was higher among mothers who were 20–39 years old as compared to those who were forty and above (B = 14.5; p = 0.001). Education level proved to be a significant predictor for the practice score. Mothers without no education had less score than mothers who had higher education levels. (B = -14.4; *P* < 0.001). Occupations and ethnicity had no effect on the practice score. However, Urban residents had better practice score compared to the ones living in rural areas (B = 6.9; *p* = 0.003). (Table 1).

## Discussion

This study provides a holistic picture depicting knowledge, attitude, and practices in mothers about newborn care, to help design evidence- based interventions in order to achieve SDG goal 3 related to newborn survival in Pakistan.

With respect to thermal protection, WHO has recommended preventive measures such as skin to skin contact, immediate placement of baby on mothers chest and delayed bathing with the gap of minimum six hours after birth are very important for a newborn as these can prevent the neonatal complication of hypothermia. The early bathing is known to be a leading risk factor for neonatal morbidity such as hypothermia and mortality [19, 20].

According to this study, 57% of the mothers had knowledge about Kangaroo care (skin to skin contact) and 57.1% practiced skin to skin contact with their babies. However, these findings are considered low when compared with a study conducted in North Ethiopia, where 99.3% of the participants had the knowledge and 72.1% practiced skin to skin contact with their babies [21]. This difference could be related to the difference in study participants. As in above-mentioned study participants were midwives because of their qualification and positions they may have a better understanding about the importance of skin to skin contact.

As far as the bathing time is concerned, in the present study, more than half (51.8%) of the respondents reported that they bathed their newborns within six hours. If compared to the study done in District *Matiari*, Pakistan, where 32% of respondents stated that they bathed their newborns within six hours, the current study’s statistics are quite high [13].

According to a study done in Nepal, cultural beliefs were related to early newborn bathing. Majority of the participants reportedly believed that early bathing of their newborns cleans the dirty coating of Vernix present on the baby. Hence, the study population has a custom of early bathing to clean and purify the baby instantly [22]. Almost the same reason can be held responsible for early bathing practices for newborns found in District Badin. Despite this, the practice percentage is much lower in Badin if compared with 78.5% people in *Gilgit,* 82% people in Karachi*,* Pakistan and 60% people in Southern Tanzania [11, 14, 23]. The difference seen here can be due to a difference in socio-cultural characteristics in different regions.

An umbilical cord is another sensitive issue concerning newborn care. WHO stresses the importance of hygiene while handling the cord and applying chlorhexidine, especially in regions where there are higher neonatal mortality rates [24]. Unhygienic deliveries and unsafe cord cutting and caring practices cause tetanus and sepsis which are two leading reasons for maternal and neonatal illnesses and deaths [19, 25].

The importance of applying Chlorhexidine was stated by Imdad A. et al. in Pakistan; he demonstrated a significant reduction in a number of cases of umbilical cord infections among newborns delivered at home [26]. In the current study, 65.4% of the respondents stated that the umbilical cord was cut by a new blade. Nearly 26.9% of respondents reported using traditional substances on the cord, whereas, only 1.5% used chlorhexidine. However, these findings were not consistent with a study conducted in urban areas of *Rohtak Haryana* located in the neighboring country India, where a new blade was used in 88.6% of the deliveries and 40% of the mothers used traditional substances on the cord [12]. The differences observed might be due to study settings and the difference in a socio-cultural background of the participants.

According to WHO, breast milk is the best way of feeding babies and supplying them with nutrients essential for their healthy growth and development [27].

Regarding the knowledge of breastfeeding, our study found out that 54.6% of the mothers knew that breastfeeding should be initiated within an hour of the baby’s birth. 57.5% knew that pre-lacteal feed should not be given to neonates and 55.6% of them stated that colostrum should be given to their newborns. These figures did not match with the ones found in a study done in Ethiopia; the respondents there had higher knowledge about breastfeeding. Around 80.9% stated in that study that breastfeeding should be initiated within the first hour of birth, 95.9% stated that pre-lacteal feed should not be given to the newborns and 96.4% stated that colostrum should be given to the newborns [28]. The possible explanation for such a stark difference in responses might be because the study in Ethiopia was conducted in urban areas as compared to this one done in rural areas that make a difference in the information about newborn care.

In this present study around half (50%) of the participants initiated breastfeeding within one hour of birth, 54.8% of them did not give any pre-lacteal feed and 55.2% administered colostrum to their newborns. This finding is higher than the findings in a study done in district *Matairi*, Pakistan which shows that breast milk was initiated within one hour by 41%, pre-lacteal feed was not given by 52.1% respondents and colostrum was given by 44% mothers [13]. This difference might be due to awareness interventions in the study community by various organizations working on child health.

According to the result of the multivariate analysis, among socio-demographic and other related factors such as maternal age, education, and place of residence had significant associations with the Knowledge and Practice score of mothers. Mothers within the age group of 20–39 had on average higher knowledge and practice score on newborn care as compared to women who were forty or above. This figure appeared to be consistent with another study done in Ethiopia [24]. The suggested possible explanation for the above result could be that mothers with young age may have better awareness regarding the benefits of newborn care practices. While older mothers may have more traditional influence or come from the traditional cohort, therefore, were less likely to have knowledge and practice in comparison to young mothers. Mothers’ education level was found to be significantly associated with the KAP of newborn care. Respondents with no formal education had lesser score than those mothers who had higher education. This finding is consistent with studies done at Tamil Nadu, in southeastern India and Kanchipuram district, India [18, 29]. A probable explanation for this finding might be that a highly educated mother could have a better understanding or awareness about the importance of newborn care. Thus, giving these mothers confidence to take the right decisions to take care of their newborns, resulting in better knowledge and accurate practices. In this study, there was no significant association of mothers’ occupation with KAP of mothers about newborn care. This finding is similar to the study done at Mandura District, Northwest Ethiopia, In which no association between mothers occupation and mothers practice of newborn care was found [30]. This inconsistency could be because of the difference in socio-demographic conditions of the respondents in the study area. Urban resident mothers were associated with the practice of newborn care than who were living in rural areas. This is in line with the two studies done in Ethiopia [30, 31]. A probable reason for this difference might be easy access to healthcare services and better education in urban areas as compared to rural areas. In our study, the socioeconomic status of the respondents had no association with the practice of newborn care. This finding is also similar to the study done in Ethiopia [31]. The reason behind this could be that health care provider disseminates information about the importance of newborn care house to house irrespective of socioeconomic status.

## Limitations of the study

Since the study is cross-sectional it may not be strong enough to demonstrate a causal relationship between dependent and independent variables due to the nature of the study. Similar studies done on KAP of newborn care are limited in our country to make comparative discussion. The information on KAP of newborn care was collected from the mothers who had given live birth during the past twelve months prior to the start of this survey. Hence, there might be some recall bias that affected the quality of data.

## Conclusion

The findings of this study showed gaps in knowledge and practice for newborn care. High-risk factors were identified in this study such as immediate bathing, application of traditional substances on the umbilical cord, delayed initiation of breastfeeding, discarding colostrum and giving pre-lacteal feed to newborns; these all need immediate attention of MNCH programs in Sindh province. Moreover, this study has also revealed many socio-demographic factors which have significant effects on the mothers’ KAP regarding newborn care. These factors included education level of mother and place of residence. Therefore, information gained from this study can be used as a baseline to provide input for developing feasible and sustainable behavioral change and educational interventions. Community-based programs are expected to address the poor practices to improve neonatal outcomes. Furthermore, it is recommended to conduct qualitative research to explore the reasons and beliefs associated with unsafe newborn care practices.

## Data Availability

The datasets used and/or analyzed during the current study are available from the corresponding author on reasonable request.

## Abbreviations

ANOVA: Analysis of variance
GLM: Generalized Linear Model
KAP: Knowledge, Attitude and Practice
MNCH: Maternal, Newborn and Child Health
SDG: Sustainable Development Goals
SES: Socio-Economic Status
WHO: World Health Organization
